# Prognostic significance of copy number variation in B-cell acute lymphoblastic leukemia

**DOI:** 10.3389/fonc.2022.981036

**Published:** 2022-08-04

**Authors:** Yang Song, Qiuyun Fang, Yingchang Mi

**Affiliations:** State Key Laboratory of Experimental Hematology, National Clinical Research Center for Blood Diseases, Haihe Laboratory of Cell Ecosystem, Institute of Hematology and Blood Diseases Hospital, Chinese Academy of Medical Sciences and Peking Union Medical College, Tianjin, China

**Keywords:** gene deletion, copy number variation, acute lymphoblastic leukemia, *CDKN2A/2B* deletion, *IKZF1* deletion, *PAX5* deletion, prognosis

## Abstract

Copy number variations (CNVs) are widespread in both pediatric and adult cases of B-cell acute lymphoblastic leukemia (B-ALL); however, their clinical significance remains unclear. This review primarily discusses the most prevalent CNVs in B-ALL to elucidate their clinical value and further personalized management of this population. The discovery of the molecular mechanism of gene deletion and the development of targeted drugs will further enhance the clinical prognosis of B-ALL.

## Introduction

B cell acute lymphoblastic leukemia (B-ALL) is a heterogeneous and invasive hematological malignancy with the accretion of genetic lesions (1, 2). Recent research has comprehensively investigated the genetic landscape of both adult and pediatric B-ALL (3–5). Over 90% of pediatric patients with B-ALL can attain complete remission (CR), 20% relapse, and 10% remain incurable (6). The conventional approach for pediatric B-ALL remission-induction chemotherapy drugs mainly consists of glucocorticoid, vincristine, asparaginase and/or anthracycline (7). With the first course of induction therapy administration for 4-6 weeks, the CR rate population of pediatric B-ALL may reach 98% (7).

The genomic pattern of adult B-ALL might differ from pediatric cases, accompanied by more devastating clinical outcomes (8). However, chances of newly emerged drugs, chimeric antigen receptor T cell therapy, and hematopoietic stem cell transplantation (HSCT) improved the clinical response of specific subtypes of B-ALL patients remarkably (9–12). Nevertheless, 40% of adult patients with B-ALL relapsed at a median duration of 13 months (28 days to 12 years) (13). In this population, around 30%-40% of relapsed and refractory B-ALL cases can attain complete remission by first salvage chemotherapy. Besides, the long-term survival, that is, the 5-year survival rate, of patients with B-ALL remains at 20% only (14, 15). Hence, it is imperative to find a novel biomarker that could help determine the characteristics and prognosis of newly diagnosed B-ALL (16, 17).

Copy number variations (CNV; a.k.a. copy number aberrations [CNAs]) are a specific type of genetic abnormality with a high incidence in B-ALL (1, 14, 18), ranging from 1 Kb to less than 5 Mb (19). CNVs denote the deletion, insertion, replication, and multipoint variants of DNA fragments. Previously, the initial cognition of CNV was found in healthy people and correlated with neuropsychiatric disorders. Today, CNV is broadly recognized as a major cause of various solid tumors (20) and acute myeloid leukemia (21). This review primarily focuses on the CNV biomarker analysis in B-ALL and their prognostic significance.

## CNV detection method

As CNVs are challenging to detect by karyotype analysis, fluorescence *in situ* hybridization (FISH), and PCR amplification; besides, their research and application are limited to some extent (22, 23). Indeed, FISH is traditionally used in CNV research but is limited to the imbalance design of both satisfying multi-genes location and the FISH gene-specific probes. With the advent of various sequencing technologies, array-based CNV analysis was commonly used for detecting genomic DNA fragments. For example, CNV can be recognized by array comparative genomic hybridization and single-nucleotide polymorphism arrays; however, the high cost and complex process of these techniques hinder their widespread use in clinical practice. In 2002, Schouten established multiplex ligation-dependent probe amplification (MLPA) assay to analyze the CNV spectrum; this technology is a fast and reliable gene CNV detection method that can detect the copy number changes of 45 gene probes simultaneously with high specificity and at a low cost (24). Kiss R et al. (25) proposed the digital MLPA-based approach based on the next-generation sequencing technology to detect hundreds of exon-positions CNV panels at the same time. The next-generation sequencing method can simultaneously detect sequence variation of a single base, insertion, or deletion of short fragments and CNV (19).

To date, many studies have investigated various software projects to examine copy number changes (26). Zhou B et al. (23) compared different sequencing depths (1×, 3×, and 5× coverages) using whole-genome sequencing by different sequencing libraries (short/3 kb/5 kb); they recommended that the gold standard for CNV detection was under the large library and low sequencing depth. Optical genome mapping is a new whole-genome sequencing method in which each DNA molecule is linearized and unfolded by nano-microfluidic CHIP with high-resolution fluorescence imaging (27, 28).

All structural variations and CNVs can be detected by providing original DNA information for downstream applications of genomics. Unlike other traditional cytogenetic methods, optical genome mapping has a full coverage of all types of mutations, detects small tumor-related mutations, and has high consistency in detecting hematological malignancies–related chromosomal and DNA abnormalities. In addition, LüHMANN JL et al. (29) established that optical genome mapping was superior to any other traditional method in the area of detecting the classical gene deletions (e.g., *IKZF1*) and gene losses that were previously undetected (e.g., SETD2). Owing to the insensitivity of whole-genome sequencing hybridization and capture, the reads captured in an exon fragment vary markedly from sample to sample. Thus, new technologies emerged gradually, such as noninvasive prenatal testing technology, which could detect CNVs in tumor circulating free DNA of 7 MB size with >95% sensitivity and specificity (30).

Reportedly, RNA-seq is limited to detect CNVs in ALL as a result of mismatching B-allele frequency. BAŘINKA et.al (31). developed a robust tool RNAseqCNV package based on the normalized gene expression and minor allele frequency to classify arm-level CNVs. In addition, InferCNV was applied widely to identify large-scale chromosomal CNVs in tumor single-cell RNA sequencing (scRNA-seq) data. The basic idea is to compare the gene expression of each tumor cell with the average expression or “normal” reference cell gene expression in the whole genome to determine its expression intensity (32). However, the genomic location of specific CNVs is not available to precisely classify tumor and normal cells copy number spectrum. Considering the critical need for distinguishing normal cell types from malignant cells in the tumor microenvironment, copy number karyotype of tuments (CopyKAT), as an integrated Bayesian segmentation method, was developed to estimate the CNV spectrum, with an average genome resolution of 5 MB from the reading depth of high-throughput scRNA-seq data (33).

## CNV prevalence in B-ALL

CNVs are frequently detected in B-ALL with considerable heterogeneity distribution (34). Overall, about 40%–49% of B-ALL carried gene CNVs that regulate early B-line cell differentiation and development-related genes (e.g., *PAX5*, *IKZF1*, and *EBF1*) and about 60% carry deletions of cell-cycle regulatory genes (e.g., *CDKN2A/B* and *RB1*) (5, 14). Broadly, CNVs occurred in 65% of pediatric B-ALL cases (35). **Table 1** summarizes the incidence of common CNVs (including *IKZF1*, *CDKN2A/B*, and *PAX5* genes) detected using MLPA from multiple cohorts (5, 18, 36–46); these occurred in the order of *IKZF1*, *CDKN2A/B*, and *PAX5*. In adult B-ALL cases, deletions of these genes were markedly enriched in the Philadelphia chromosome-positive (Ph+) B-ALL group than in the Ph− B-ALL group (82.4% vs. 58.7%, *P<0.01*) *(* 18). Furthermore, Ribera J et al. (47) detected CNAs of 12 genetic regions in 142 adolescents and adults with *de-novo* precursor B-ALL using MLPA; *CDKN2A/B* deletion occurred in 59/142 (42%) cases, while *IKZF1* deletion occurred in 49/142 (35%) cases.

**Table 1:** CNVs in frequent genes in different B-ALL cohort.

	Author/Group	Subtype	Patient number	B-ALL status	IKZF1N (%)	CDKN2A/2B N (%)	PAX5	IKZF1 ^plus^(%)	No Del(%)	Reference
							N (%)			
Pediatrics	ALL IC-BFM 2009	Whole series	88	ND	16(18.2%)	23(26.1%)	(30.7%)	(12.5%)	(35%)	(18)
						(25%)				
	UKALL14	Whole series	437	ND	170(38.9%)	162(37.1%)	93(21.2%)	–	167(38%)	(36)
	MIGICCL study	Whole series	63	ND	17(27%)	20(31.7%)	10(15.9%)	–	25(39.7%)	(37)
						18(28.6%)				
	Hamadeh L et al.	Whole series	3239	ND	12%	30%	20%	–	42%	(38)
	NOPHO protocols	Ph-	116	ND	19(16%)	47(41%)	40 (35%)	–	–	(39)
	Gupta SK et al.	Ph^-^	320	ND	47(14.7%)	103(32.2%)	82(25.6%)	32(10%)	141(44%)	(40)
Adult	Pfeifer H et al.	Ph+	97	ND	72(74%)	41(42%)	39(40%)	–	–	(41)
	GIMEMA protocols	Ph+	116	ND	97(84%)	30(32%)	43(36.2%)	45(46.4%)	–	(42)
	Chiaretti S et al.	Ph+	60	ND	84.6%	33.3%	38.5%	–	21%	(43)
	Fang Q et al.	Ph+	85	ND	65.9%	28.2%	27.1%	30.5%	17.6%	(5)
		Ph-	126	ND	20.6%	42.1%	23.8%	15.08%	41.3%	
	Dirse V et al.	Whole series	66	ND	4(6%)	19(29%)	4(6%)	–	–	(44)
						18(27%)				
	Roberts KG et al.	Ph like	165	ND	120(73%)	84(51%)	62(38%)		14%	(45)
	GIMEMA LAL1913	Non-Ph like	48	ND	12(25%)	23(47.9%)	11(22.9%)	7(14.6%)	–	(46)
		Ph like	22	ND	14(63.6%)	7(31.8%)	7(31.8%)	10(45.5%)	–	

Annotation: ND, newly diagnosis.

Nevertheless, the research on CNV clones in relapsed B-ALL is limited. Despite being the preferred and widely used method for detecting CNVs in the related literature, MLPA might not be able to detect CNVs in samples presenting a low leukemia burden (carried <25% CNV clone). Moreover, CNVs in relapsed B-ALL remain unclear owing to limited paired B-ALL (newly diagnosed and relapsed) samples. The CNVs of relapsed B-ALL evolved from the diagnosis for examining specific gene content and clone size. By comparing the first-relapsed B-ALL to the newly diagnosed stage. RIBERA J et al. (48) established that *CDKN2A/B*, *PAX5*, and *IKZF1* deletions were more frequent at relapse. Mullighan CG et al. (49) performed the genome-wide CNV and LOH analyses on matched diagnostic and relapse bone marrow samples from 61 pediatric patients with ALL, and identified a mean of 10.8 somatic CNV per B-ALL case and 7.1 CNVs per T-ALL case at diagnosis. In addition, they observed a significant increase in the mean number of CNVs per case in relapsed B-ALL samples (10.8 at diagnosis vs. 14.0 at relapse, *P* = 0.0005); however, no significant changes were observed in the lesion frequency in T-ALL. The majority (88.5%) of relapse samples harbored at least some of the CNAs present in the matched diagnosis sample, suggesting a common clonal origin, although 91.8% of samples showed a change in the pattern of CNVs from diagnosis to relapse. Of these cases, 34% acquired new CNVs, 12% exhibited loss of lesions present at diagnosis, and 46% both acquired new lesions and lost lesions present at diagnosis. Moreover, Ribera (48) compared CNVs at diagnosis and relapse, observing the trend to acquire homozygous *CDKN2A/B* deletions and a considerable increase in CNVs from diagnosis to the first relapse. Besides, evolution from an ancestral clone was the main pattern of clonal evolution. When focusing on the acquired CNVs in relapsed clones, gene alterations mostly correlated with proliferation and drug resistance.

## Clinical significance of recurrent CNV genes in B-ALL

### *IKZF1* gene deletions

The Ikaros Zinc Finger 1 (*IKZF1*) gene, located at 7p12.2, encodes 519 amino acids by 8 full-length exons (50). Exons are essential for Ikaros gene functions, except for exon 1 (which does not participate in transcription) and exons 2, 3, and 7 (undetermined significance). *IKZF1* deletions in both coding and noncoding regions might interfere with the gene activity and promote B-ALL progression through specific targets. For example, *EBF1*, *MSH2*, and *MCL1* genes, as the target genes of *IKZF1*, play a vital role in affecting B-cell differentiation (*EBF1* gene), DNA repair (*MSH2* gene), and anti-apoptosis (*MCL1* gene). The primary functions of the *IKZF1* gene include B-cell differentiation blocking, metabolic reprogramming, leukemia microenvironment adhesion, disease relapse, and drug resistance (51).

Increasing evidence indicated that *IKZF1* deletions mediate cellular drug resistance and relapse. For example, Rogers et.al (52) established that the *IKZF1* deletion was resistant to dexamethasone, asparaginase, and daunorubicin by upregulating the JAK/STAT pathway. In addition, the *IKZF1* deletion affects sensitivity to cytarabine by downregulating the SAMHD1 pathway (52); STEEGHS et. al (14) suggested that the loss of *IKZF1* caused prednisolone resistance by elevating intracellular ATP and glucose levels, whereas drug sensitivity was recovered by inhibition of glycolysis. Moreover, *IKZF1* deletion events, accompanied by CREBBP deletion or mutation, were common in relapsed pediatric B-ALL patients, which could correlate with the selective pressure of chemotherapeutic drugs on tumor cells (8).

Notably, *IKZF1* gene deletions comprise localized large fragment deletions, single exon deletions, and other nonlocalized deletions, among which localized large fragment deletions are the most common. The loss of *IKZF1* can be separated depending on its functional effect. While IK1–IK3 is considered a functional subtype, other subtypes are dominant-negative isoforms (DN isoforms), that is, functional defect subtype. In addition, IK6, often located in the cytoplasm, is a functional defect subtype with the complete loss of N-terminal zinc finger structure due to exon 4–7 deletion. IK6 functions as DN effects by isolating normal cytoplasmic proteins (53). Loss-of-function was designated as the total allelic inactivation. The loss of haploid dysfunction due to exon 2 deletion can decrease the Ikaros protein level.

Some studies reported *IKZF1* deletions in around 15% of pediatric B-ALL cases and 30%–40% of adult B-ALL cases (40, 54). Perhaps, *IKZF1* deletions in pediatric B-ALL are a hallmark of high-risk stratification and relapse independently carried by 70% of high-risk pediatric B-ALL (45, 55, 56).

In adult B-ALL, *IKZF1* deletions were detected about 70% of Ph+ B-ALL cases (4, 5), around 15%–30% of Ph− B-ALL (53), and 40% of Ph-like B-ALL cases (13). In a study, *IKZF1* deletions were mostly enriched in the adult Ph+ B-ALL group than in the Ph− B-ALL group (65.9% vs. 20.6%, *P* < 0.01) (5). Ribera reported that *IKZF1* deletions were more prevalent in Ph+ B-ALL (52%) and correlated with advanced age and high white blood cell count (47, 57). Another study reported that *IKZF1* deletions correlated with the *CALF2* gene overexpression (*P* = 0.001), particularly in DN isoforms (*P* = 0.006), regardless of age (54). Furthermore, *IKZF1* deletions with *CRLF2* overexpression indicated a poor prognosis in both adult and pediatric B-ALL patients (54).

The prognostic impact of *IKZF1* alterations in B-ALL remains debatable (58).

Kobitzsch (53) reported that loss-of-function not DN intragenic *IKZF1* deletions correlated with an adverse prognosis in adult BCR-ABL-negative ABL. Yeoh AEJ et al. (59) compared the 5-year cumulative incidence of relapse (CIR) of Malaysia–Singapore MS2003 (*n* = 507) and MS2010 (*n* = 316) of pediatric B-ALL; the findings revealed that the loss of *IKZF1* strongly correlated with a higher 5-year CIR (20.5% vs. 8.0%, *P* = 0.01) in MS2003. However, the treatment of *IKZF1* deletion patients was intensified in MS2010, and the 5-year CIR presented no more significant difference in pediatric Ph− B-ALL (11.4% vs. 4.4%, *P* = 0.09). In addition, Ribera reported that *IKZF1* deletions conferred a higher relapse incidence (40% vs. 58%, *P* = 0.048) and worse 5-year overall survival (OS; 29% vs. 50%, *P* = 0.023) than *IKZF1* undeleted in Ph− B-ALL (47). Zhang W et al. (58) conducted a meta-analysis of the correlation between *IKZF1* deletion and survival; *IKZF1* lesions could independently predict unfavorable OS (hazard ratio [HR] 1.60, 95% confidence interval [CI] 1.25–2.06) and event-free survival (EFS; HR 1.67, 95% CI: 1.28–2.17) in Ph− B-ALL. In the EsPhALL cohort (pediatric BCR-ABL1-positive), *IKZF1* deletions correlated with an unfavorable prognosis (4-year Disease Free Survival [DFS] of 51.9% ± 8.8% for *IKZF1*-deleted vs. 78.6% ± 13.9% for *IKZF1* wild-type; *P* = 0.03). The massive analysis of *IKZF1*-loss patients demonstrated that it played a crucial role in Ph-like B-ALL. In ALL-BFM protocols, *IKZF1* deletions acted as an independent risk factor, with the lower 5-year EFS than wild-type *IKZF1* (0.69% vs. 0.85%, *P* < 0.0001) (51). Furthermore, *IKZF1* deletions in Ph-like B-ALL multivariate models could precast EFS and OS (60, 61).

The response of early chemotherapy induction in patients with *IKZF1* deletions was disappointing over the whole series. Several studies established that patients with *IKZF1* lesions exhibited a high minimal residual disease (MRD) level (51, 60, 62). Reportedly, these patients could benefit more from intensive/alternate therapy than standard ones (4). Reportedly, the combination of vincristine and steroids in patients with *IKZF1* deletions during maintenance treatment could be an effective and reasonable approach to prevent relapse. Dhédin N et al. (63) demonstrated that patients with *IKZF1* deletions were likely to benefit from allogeneic HSCT (allo-HSCT) in terms of EFS (HR 0.42, 95% CI: 0.18–1.07, *P* = 0.025) and OS (HR 0.35, 95% CI: 0.16–0.75, *P* = 0.007), compared with non-*IKZF1* alteration groups in adult Ph− B-ALL populations. However, whether the poor prognosis of *IKZF1* overcame by stem cell transplantation warrants further investigation.

### CDKN2A/CDKN2B gene deletion

Cyclin-dependent kinase inhibitor 2A/B (*CDKN2A/B*) is a common deletion in pediatric and adult B-ALL CNV profiles (1, 57, 64), as well as a major proposition of E2A-PBX1–positive B-ALL, but limited in MLL-rearranged patients (*P* = 0.005) (65). *CDKN2A/B* deletion is the major suppressor gene CNV in chromosome 9p21 (66). Compared with children, the *CDKN2A/B* incidence rate is marginally higher in adults (*P* = 0.002) (67). Moreover, 24.6% (14/57) of Ph-like patients present with enriched biallelic loss of *CDKN2A/B* (68). Reportedly, this lesion was highly representative of high white blood cell count, older age at initial diagnosis, and often accompanied by *IKZF1* deletions (called I&C) (36, 69). Remarkably, clones with *CDKN2A/B* deletions detected in the initial diagnosis always persisted in relapse cases. Furthermore, *CDKN2A/B* presented a notable increase in the CNVs of relapse B-ALL (48).

In some studies, pediatric B-ALL patients with *CDKN2A/B* deletions exhibited a trend of shorter relapse time and EFS (35, 67), although the OS rate remains debatable. Kathiravan et. al (35) indicated that the 28-month EFS of *CDKN2A/B* lesions in ICICLE (Indian adaption of UKMRC2007 protocol) was notably decreased (42% vs. 90%, *P* = 0.0004) compared with non-*CDKN2A/B* deletions. Moreover, Braun M et al. (69) proved that CDKN2A deletions decreased the RFS significantly (HR 2.21, *P* = 0.028). No evidence indicates that loss of *CDKN2A/B* affected the prognosis in pediatric EORTC trials (70). Conversely, Feng J et al. (71) suggested that *CDKN2A/B* deletions inferred the 3-year EFS rate (69.8% vs. 89.2%, *P* = 0.000) and 3-year OS rate (89.4% vs. 94.7%, *P* = 0.037).

The frequency of adult *CDKN2A/B* deletions in the Ph-B-ALL group was much higher than in the Ph+ B-ALL group (39.7% vs. 24.7%, *P* = 0.041) (5). The prognostic value of *CDKN2A/B* in adults has been debated previously (35, 41, 44). Most studies emphasized that *CDKN2A/B* did not affect EFS and OS of adult patients with B-ALL. Only a few studies emphasized that *CDKN2A/B* adversely affected adult patients with B-ALL. Fang et. al (72). reported that *CDKN2A/B* is the vital relapsing and inferior prognostic marker for adult Ph− B-ALL (2-year OS: 38.2% vs. 80.3%, *P* = 0.002; 2-year RFS: 44% vs. 88.9%, *P* = 0.006). Messina M et al. (73) enrolled B-ALL-negative patients for BCR-ABL1 (Ph− B-ALL) population, including children, adolescents, and adults; the *CDKN2A/B*/*RB1* deletion was reported as the negative prognostic factor (HR 2.12, *P* = 0.048) regardless of age. Pfeifer H et al. (41) suggested that *CDKN2A/B* deletions played an independent prognostic role in predicting the risk of relapse (DFS HR 2.621, *P* = 0.0054) and OS (HR 2.162, *P* = 0.014) in the adult Ph+ B-ALL population. Moreover, Dirse et. al (44). reported that *CDKN2A/B* decreased the EFS (multivariate HR 2.607, *P* = 0.034) in the whole series of adult B-ALL.

### *PAX5* gene deletion

The transcription factor paired box domain gene 5 (*PAX5*) was considered to regulate B-cell lineage differentiation and contribute to leukemogenesis in B-ALL (74, 75). *PAX5* acts on the downstream transcription factors E2A and EBF1 and is crucial for B-line differentiation (76). In *PAX5*-deficient mice, the development of B cells in the bone marrow was blocked in the early Pro-B stage (77). The alterations of *PAX5* comprise partial exon deletion on chromosome 9 (14%) and amplification of exon 2 or 5, resulting in frameshift mutation (7%). *PAX5* deletions might increase genetic instability. Consequently, the probability of a secondary strike markedly increases and induces the recurrence and development of leukemia. In a study, *PAX5* deletions decreased leukemia cell viability by inducing apoptotic cell death using a new ribozyme-derived isotype-specific knockdown system in the B-ALL cell model (77). Furthermore, transplantation experiments and exhaustive sequencing validated that *PAX5* deletion made it sensitive to malignant transformation by forming an abnormal progenitor cell population (78).

As shown in **Table 1**, *PAX5* deletions occurred in 15.9%–31.7% of pediatric Ph− B-ALL, 33% of pediatric Ph+ B-ALL (14), 27.1%–40% of adult Ph+ B-ALL, and 22.9%–23.8% of adult Ph-B-ALL (31.8%–38% Ph-like ALL) cases. No statistical difference has been reported between adult Ph− B-ALL and Ph+ B-ALL (27.1% vs. 27.8%, *P* = 0.549) cases (5). Most *PAX5* deletions coexisted with *CDKN2A/B* deletions (83.3% of children and 100.0% of adults) and were commonly deleted in *ETV6-RUNX1* B-ALL. The prognostic significance of *PAX5* deletions in adult B-ALL also remains debatable. BHANDARI P et al. (64) claimed that *PAX5* deletions were unsuitable for an independent prognostic marker for predicting prognosis because of no significant influence of RFS among B-ALL subgroups (*P* = 0.6839). Moreover, Iacobucci I et al. (79) reported no correlation between *PAX5* deletions and OS (*P* = 0.3294) or DFS (*P* = 0.9249) in adult Ph+ B-ALL. In contrast, FEDULLO AL et al. (42) suggested that adult Ph+ B-ALL with *PAX5* deletions showed shortened DFS (24.9% vs. 43.3%; *P* = 0.026).

In pediatric B-ALL groups, the prognosis of *PAX5* deletion was strongly dependent on *IKZF1* codeletion (61, 80). However, no significant prognostic correlation was observed in *PAX5* deletions alone in children (74). In other words, the *PAX5* -loss group presented no relapsing risk after excluding *IKZF1* deletions. Indeed, double deletion of *PAX5* and *IKZF1* was improved by treatment intensification in MS2010, with 0% 5-year CIR than 80.0% in MS2003 (*P* = 0.05).

## Prognostic relevance of integrated CNV profiling

Extensive research integrated gene CNV profile into pediatric B-ALL risk stratification (17). Moorman AV et al. (34) identified an 8-gene CNV panel, including *IKZF1*, *CDKN2A/B*, *PAR1*, *BTG1*, *EBF1*, *PAX5*, *ETV6*, and *RB1*, for stratifying the pediatric B-ALL risk level known as the UKALL-CNV classifier (**Table 2**). This tool has robust decision-making ability in intermediate-risk cytogenetics subgroups and even patients with different leukemia protocols baseline (37, 38). Besides, the UKALL-CNV classifier can refine the established cytogenetic risk groups.

**Table 2:** Current stratification classifier of pediatric B-ALL CNV profile.

Classifier	Group	Content	Survival
Moorman risk criteria (UKALL2003) (34)	CNV Good Risk (CNV-GR)	Isolated allelic losses of ETV6, PAX5, BTG1 ETV6 deletions with a single additional loss of BTG1, PAX5, CDKN2A/B; Absence of any deletion of IKZF1/*CDKN2A/B/PAX5/ETV6/BTG1/EBF1/RB1/PAR1.*	MIGICCL Study (37) DFS (82% vs. 33% vs. 38%, *p* <0.0001) OS (65% vs. 5% vs. 44%, *P = 0.005*)
	CNV Poor Risk (CNV-PR)	Any single deletion in *IKZF1, RB1, PAR1* or *EBF1*; the combined loss of *IKZF1/PAX5/CDKN2A/B.*	
	CNV Intermediate Risk (CNV-IR)	Patients with none of those and/or another alteration profile.	
Hamadeh L et al. (38)	Gen-VGR	Cyto-GR+ CNV-GR	EFS (91% vs. 81% vs. 73% vs. 54%, P < 0.001)
	Gen-GR	Cyto-IR+CNV-GR; Cyto- GR+ CNV- IR	
	Gen-IR	Cyto-IR+CNV-IR/CNV-PR; Cyto-GR+CNV-PR	
	Gen-PR	Cyto-HR, regardless of CNV	
*IKZF1* ^plus (^STANULLA M et al.)	IKZF1^plus^ present	*IKZF1* deletion + any deletion of *CDKN2A*, *CDKN2B*, PAX5 or PAR1(score IKplus1)	5-year EFS (53 ± 6% vs. 79 ± 5%, P < 0.001)
(81)	IKZF1^plus^ absent	IKZF1^plus^ absent (score IKplus0)	Adult GIMEMA LAL2116 protocol (82) DFS (84.5% vs. 54.5%, *P = 0.026*) Adult GIMEMA LAL1509 protocol (43) DFS (0% vs. 60% P = 0.0008); OS (20% vs. 69.5%, *P = 0.0068*)
MRplus (40)	Good-risk	MRplus0= score M0 + score IKplus0	Post-induction remission response (90.7% vs. 77.8% vs. 73.9%, *p = 0.004*)
	Intermediate- risk	MRplus1= scoreM1+score IKplus0	EFS (56% vs. 34% vs. 19%, *p < 0.001*)
	Poor-risk	MRplus2= score M1+ score IKplus1	
Moorman Revised (UKALL14) (36)	Very high risk (VHR)	CK, HoTr or JAK-STAT abnormalities	OS VHR vs. SR 27% vs.64%, *P<0.001* HR vs. SR 45% vs.64%, *P = 0.013* TKA vs. SR 57% vs. 64%, *P = 0.107*
	High risk (HR)	KMT2A fusions (*KMT2A-AFF1)*	EFS
	Standard risk (SR)	BCR-ABL1 and ABL class fusion	VHR vs. SR 23% vs.58%, *P<0.001* HR vs. SR 37% vs.58%, *P = 0.008* TKA vs. SR 47% vs. 58%, *P = 0.005*
	Tyrosine kinase activating (TKA)	All other patients	

Annotation: Cyto-GR includes ETV6-RUNX1 and high hyperdiploidy (51-65 chromosomes); Cyto-PR includes t(9;22)(q34;q11.2)/BCR-ABL1, MLL translocations, near haploidy (<30 chromosomes), low hypodiploidy (30-39 chromosomes), intrachromosomal amplification of chromosome 21 (iAMP21), or t(17;19)(q23;p13)/HLF-TCF3; Cyto-IR includes all other cases with abnormal or normal cytogenetics.

VER, very early relapsed; HR, Hazard Ratio.

The definition of CNV-GR, CNV-IR and CNV-PR was equal to the Moorman risk criteria content respectively. Gen-VGR, Gene very good risk; Gen-GR, gene good risk; Gen-IR, gene. Cyto GR, cytogenetic good risk; Cyto IR, cytogenetic intermediate risk; Cyto PR, cytogenetic poor risk.

The definition of score M0 in MRplus refers to the Low genetic risk in Moorman risk criteria score. Score M1 refers to the High genetic risk and Intermediate genetic risk in Moorman risk criteria score.

The definition of score IKplus0 and IKplus1 in MRplus refers to the IKZF1 ^plus^ absent and IKZF1 ^plus^ absent respectively. In Moorman Revised UKALL14, HoTr, low hypodiploidy/near triploidy; CK, complex karyotype ≥5 chromosomal abnormalities.

Based on the Moorman’s criteria, Gupta SK et al. (83) subgrouped the MRD-negative intermediate-risk pediatric Ph− B-ALL into two subgroups with different EFS (77% vs. 38%, *P* = 0.045) and OS (90% vs. 30%, *P* = 0.037), whereas the criteria had no classifying power in MRD-positive groups (OS 75% vs. 57%, *P* = 0.293). A total of 3239 pediatric B-ALL cases were applied to validate the UKALL classifier (38). By integrating CNV and cytogenetic data, Hamadeh revised the overall genetic classification by defining four risk groups with distinct EFS rates (*P* < 0.001)—very good (91%), good (81%), intermediate (73%), and poor (54%). Stanulla M et al. (81) proposed a very-poor prognostic subtype defined as *IKZF1* ^plus^ subtype: *IKZF1* occurred with additional mutations, containing *CDKN2A*, *CDKN2B*, *PAX5*, or *PAR1* deletions simultaneously but without *ERG* deletions (**Table 2**). Besides, the *IKZF1* ^plus^ 5-year EFS rate in pediatrics was 53% ± 6% compared with 79% ± 5% in adults (*P* < 0.001).

In adult Ph+ B-ALL, *IKZF1* ^plus^ negatively affected the survival outcome than *IKZF1* alone (DFS: 43.3% vs. 24.9%, *P* = 0.026; OS: 62.6% vs. 40.2%, *P =* 0.02)  (42). Reportedly, *IKZF1* ^plus^ patients had been under similar conditions in the GIMEMA LAL2116 cohort (DFS: 84.5% vs. 54.5%, *P* = 0.026) and GIMEMA LAL1509 protocol (DFS: 0% vs. 60%, *P* = 0.0008; OS: 20% vs. 69.5%, *P* = 0.0068) (43, 82). However, the prognostic significance of *IKZF1* ^plus^ in adult Ph^+^ B-ALL was not detected (5). Likewise, Chiaretti S et al. (46) reported no statistical correlation between *IKZF1* ^plus^ in adult Ph-like B-ALL (HR 1.869, 95% CI: 0.49–6.67, *P* = 0.339). In addition, GUPTA SK et al. (40) proposed the “MRplus” risk score system by integrating *IKZF1* ^plus^ and the UKALL-CNV classifier subtyping to better classify pediatric Ph− B-ALL prognosis. The 0, 1, 2 groups defined by MR plus system markedly discriminated postinduction remission response and 4-year OS (**Table 2**).

Considering the primary chromosomal abnormalities significantly correlate with the CNV frequency, Moorman AV et al. (36) revised the stratification by adding cytogenetic risk factors, like *KMT2A* fusions, complex karyotype and low hypodiploidy/near-triploidy. The new risk system could predict the 3-year OS (64% vs. 47%; HR 1.65 95% CI: 1.27–2.12, *P* < 0.001).

## Future perspectives

Many studies have proved that CNV is a common molecular abnormality in the development of B-ALL (48). Current evidence suggests that the CNV pattern of adult and pediatric B-ALL has a different cytogenetic abnormality and pathological significances. Moreover, growing evidence indicates that high number and diverse CNVs observed are acquired in the process of disease relapsing (37). This study mainly discussed the clinical significance of the CNV spectrum, which has been well recognized in patients with B-ALL. Among them, *IKZF1*, *CDKN2A/B*, and *PAX5* are the leading prevalent gene alterations in B-ALL (47). Moreover, these CNVs in Ph-like and Ph+ B-ALL remain equally frequent (68). However, some research of gene prognostic value is inconsistent, which could be because of difference in enrolled patients and treatment regimen. Undoubtedly, CNVs guided the risk of relapsing and survival outcome of both pediatric and adult B-ALL (84). Intensive chemotherapy combined with allo-HSCT is expected to overcome the adverse impact of CNVs. Perhaps, the combination of intensive chemotherapy and allo-HSCT could overcome the adverse impact of CNVs.

Typically, the risk stratification of B-ALL based on the CNV profiles is largely limited to the pediatric population (36). Currently, the *IKZF1* ^plus^ and UKALL-CNV classifier are broadly promoted in the adult B-ALL classification (5, 37, 43). Considering the different cytogenetic patterns of adults and children, the risk system in adults warrants revision in future. With the new exploration of new targets of rearrangement in B-ALL (e.g., *DUX4*, *ZNF384*, and *MEF2D*), the survival risk stratification system will be consistently updated in the future. Besides, further research will help identify new prognostic indicators and potential therapeutic targets.

In conclusion, this review characterizes B-ALL–related copy number events, which is valuable for precise patient subgroup stratification. In addition, this study provides insights into the new immunotherapy-based approaches and tailored treatment strategies for patients with B-ALL. Nevertheless, additional multicenter survival data will be needed for further verification in the future.

## Publisher’s note

All claims expressed in this article are solely those of the authors and do not necessarily represent those of their affiliated organizations, or those of the publisher, the editors and the reviewers. Any product that may be evaluated in this article, or claim that may be made by its manufacturer, is not guaranteed or endorsed by the publisher.

## Author contributions

YS drafted the manuscript. QF and YM revised the manuscript. All authors read and approved the final manuscript.

## Funding

Basic Scientific Research Project of National Universities (332021059).

## Conflict of interest

The authors declare that the research was conducted in the absence of any commercial or financial relationships that could be construed as a potential conflict of interest.

## Publisher’s note

All claims expressed in this article are solely those of the authors and do not necessarily represent those of their affiliated organizations, or those of the publisher, the editors and the reviewers. Any product that may be evaluated in this article, or claim that may be made by its manufacturer, is not guaranteed or endorsed by the publisher.
